# Disparity in endometriosis diagnoses between racial/ethnic groups

**DOI:** 10.1111/1471-0528.15805

**Published:** 2019-05-21

**Authors:** LV Farland, AW Horne

**Affiliations:** ^1^ Department of Epidemiology and Biostatistics Mel and Enid Zuckerman College of Public Health University of Arizona Tucson Arizona USA; ^2^ MRC Centre for Reproductive Health Queen's Medical Research Institute University of Edinburgh Edinburgh UK

Bougie and colleagues systematically reviewed the current literature on the association between race/ethnicity and endometriosis diagnosis (Bougie et al. *BJOG* 2019;126:1104–15). Compared with white women, black women were less likely (OR 0.49, 95% CI 0.29–0.83; *n* = 16 studies) and Asian women were more likely (OR 1.63, 95% CI 1.03–2.58; *n* = 10 studies) to be diagnosed with endometriosis. The data suggest that compared with white women, Hispanic women were less likely to be diagnosed with endometriosis, with a similar magnitude of effect as black women, but this finding was not statistically significant (OR 0.46, 95% CI 0.14–1.50; *n* = 5 studies). There are many unanswered questions regarding the interpretation of these findings for research and clinical practice. Ultimately, we do not know whether these associations are artifacts of diagnostic biases or whether there is heterogeneity in endometriosis phenotype or clinical presentation between racial/ethnic groups.

Endometriosis has historically been described as a disease of affluent, high‐achieving women with private health insurance who have delayed marriage and childbearing (Meigs *Obstet Gynecol* 1953;2:46–53). These early descriptions of endometriosis patients implied a rarity of disease in the ‘non‐private patient and, therefore by inference (often stated as such) uncommon in black women’ (Chatman *Am J Obstet Gynecol* 1976;125:987–9), despite early research showing no difference between black and white women and suggesting that pelvic pain in black women was misdiagnosed (Chatman *Am J Obstet Gynecol* 1976;125:987–9). In 1979, Buttram wrote, ‘Typically, our patients with endometriosis appear to have an intense desire to excel. They are usually well‐dressed and have trim figures’ (Buttram, Jr *Fertil Steril* 1979;31:117–23). Although overtly sexist, classist and racist descriptions of patients with endometriosis are less common today, these not‐so‐distant views may still consciously or unconsciously influence clinical care, leading to disparities in endometriosis diagnoses.

Differences in endometriosis diagnoses between black, Asian, Hispanic, and white women are likely to be synergistic with larger systemic problems related to the difficulty of receiving an endometriosis diagnosis. Indeed, women globally wait, on average, 7 years between the onset of endometriosis symptoms and disease diagnosis, with some women never receiving a formal diagnosis. There are numerous factors that contribute to this diagnostic delay, including inconsistent symptom recognition by both the patient and the provider, and the lack of a non‐invasive diagnostic test. When the results of the present study were restricted to women presenting with infertility, disparities for black women attenuated and there was no statistically significant difference in endometriosis diagnoses between black, white, and Asian patients, suggesting that once patients establish access to clinical care, disparities in diagnosis diminish.

Bougie et al. described substantial heterogeneity (*I* ^2^ > 50%) between studies. Although the authors stratified by study year and diagnosis modality, differences in access to health care across populations are also likely to contribute to this heterogeneity, given the social and economic complexities of diagnosis. Different countries will have different medical referral patterns and insurance systems, as well as different racial and economic biases. Both race and gender have been shown to unconsciously influence referral patterns and clinical care (Schulman et al. *N Engl J Med* 1999;340:618–26). Although future studies investigating the association between race/ethnicity and endometriosis are warranted, they must be interpreted through the lens of quantifying disparities in access to diagnostic care. We must ensure that women of all racial and ethnic backgrounds are comfortable recognising and describing their symptoms, and that healthcare providers take these symptoms seriously and provide appropriate and timely referral for endometriosis diagnosis and treatment.

## Disclosure of interests

AWH receives grant funding from the National Institute for Health Research (NIHR), the Medical Research Council (MRC), the Chief Scientist's Office, Wellcome Trust, Wellbeing of Women, Ferring, and Roche. He has received honoraria for consultancy for Ferring, Roche, and Abbvie. Dr Farland has nothing to disclose. Completed disclosure of interests form available to view online as supporting information.

## Funding

AWH is supported by funding from MRC Centre Grant (MR/N022556/1).
